# How age and infection history shape the antigen‐specific CD8^+^ T‐cell repertoire: Implications for vaccination strategies in older adults

**DOI:** 10.1111/acel.13262

**Published:** 2020-10-20

**Authors:** Josien Lanfermeijer, José A. M. Borghans, Debbie van Baarle

**Affiliations:** ^1^ Center for Infectious Disease Control National Institute for Public Health and the Environment Bilthoven the Netherlands; ^2^ Center for Translational Immunology University Medical Center Utrecht the Netherlands; ^3^ Virology & Immunology Research Department of Medical Microbiology and Infection prevention University Medical Center Groningen the Netherlands

**Keywords:** aging, CD8+ T‐cell, infection history, repertoire, T‐cell receptor, vaccination

## Abstract

Older adults often show signs of impaired CD8^+^ T‐cell immunity, reflected by weaker responses against new infections and vaccinations, and decreased protection against reinfection. This immune impairment is in part thought to be the consequence of a decrease in both T‐cell numbers and repertoire diversity. If this is indeed the case, a strategy to prevent infectious diseases in older adults could be the induction of protective memory responses through vaccination at a younger age. However, this requires that the induced immune responses are maintained until old age. It is therefore important to obtain insights into the long‐term maintenance of the antigen‐specific T‐cell repertoire. Here, we review the literature on the maintenance of antigen‐experienced CD8^+^ T‐cell repertoires against acute and chronic infections. We describe the complex interactions that play a role in shaping the memory T‐cell repertoire, and the effects of age, infection history, and T‐cell avidity. We discuss the implications of these findings for the development of new vaccination strategies to protect older adults.

## Graphical abstract

In this review, we aim to provide insight in the maintenance of antigen‐experienced CD8+ T‐cell clones during life. We describe what is already known on the effect of age on the virus‐specific T‐cell repertoire and discuss the contribution of infection history in shaping the repertoire. Insight in factors shaping the T‐cell repertoire will help to improve the protective memory response against infectious diseases in older adults.

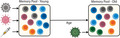

## 1 INTRODUCTION

Cytotoxic CD8^+^ T cells are important in the clearance of virus‐infected cells and are therefore of interest for the development of vaccination strategies against infectious diseases. An important correlate of protection against infectious diseases is the recruitment of T cells carrying high‐affinity antigen‐specific T‐cell receptors (TCRs) (Tscharke et al., 2015). The diversity of the recruited T‐cell repertoire is also directly linked to disease outcome (Price et al., 2004, 2009), and narrow T‐cell repertoires are associated with more frequent occurrence of viral escape (Cornberg et al., 2006). Higher TCR diversity has been suggested to be important for a protective immune response, as it increases the chance of both high avidity clones as well as cross‐reactive clones, able to recognize antigen variants, to be present in the responding repertoire (Nikolich‐Zugich et al., 2004; Turner et al., 2009).

**Figure 1:**
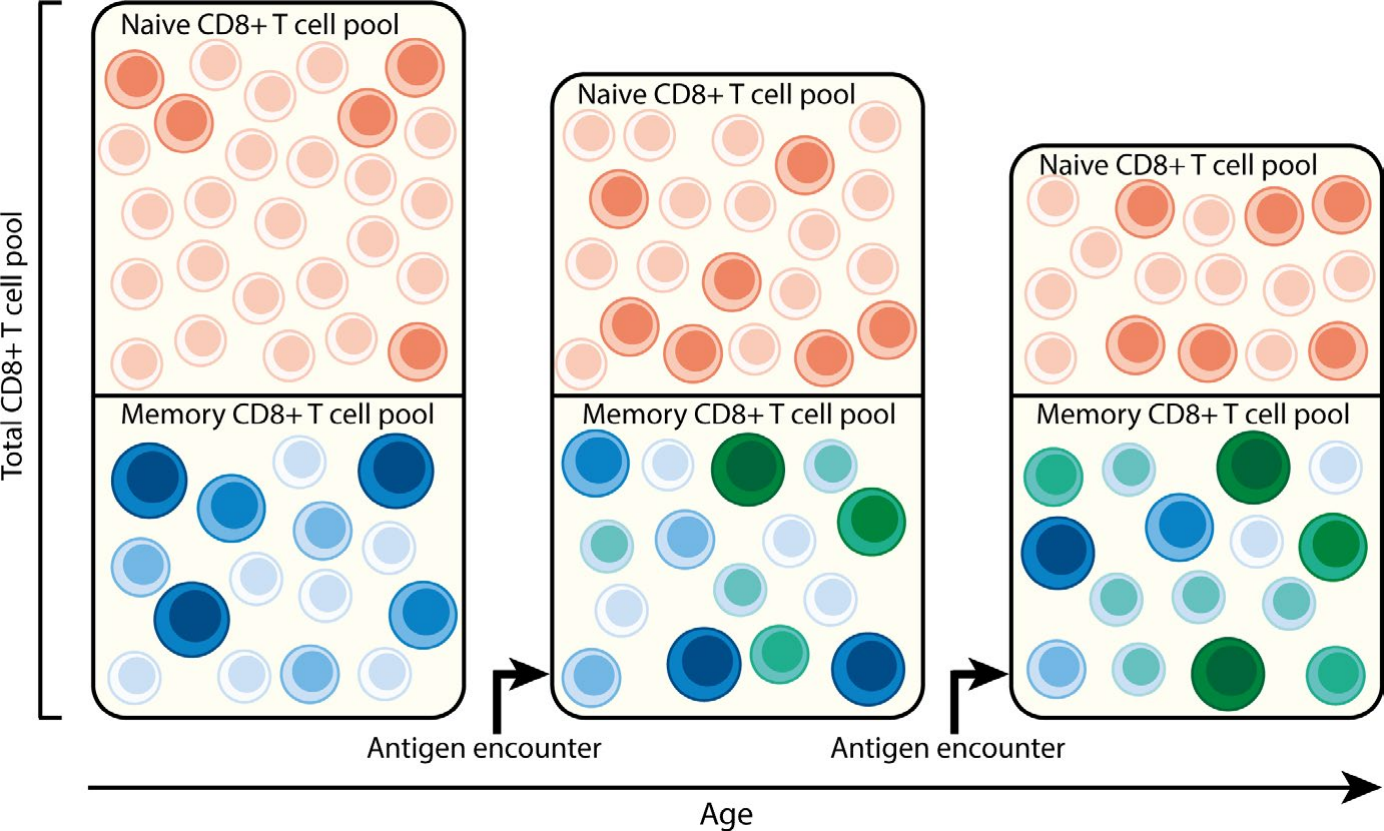
Influence of age on the human CD8^+^ T‐cell repertoire. Every circle represents a distinct T‐cell clone, the larger the circle, the larger the clonal size. The diversity of the CD8^+^ T‐cell repertoire decreases with age. This is mostly due to a decrease in the size of the naive T‐cell pool (shown in red). As the size and diversity of the memory T‐cell pool (shown in blue) remains relatively stable during adult life (Qi et al., 2014), despite the fact that new memory T cells (shown in green) are continuously added to the memory T‐cell pool, this may lead to displacement of early induced memory T‐cell clones

Diminished CD8^+^ T‐cell responses to new infections and vaccination in older adults are thought to be due to a combination of decreased numbers and functional capacity, among which the priming capacity, of naive T cells with age (Briceno et al., 2016). Another important role is assigned to a decline in the diversity of the T‐cell repertoire with age (Nikolich‐Zugich, 2008). Although estimating T‐cell diversity is challenging for various reasons (Box 1), the diversity of the total CD8^+^ T‐cell pool has indeed been shown to decrease with age in both longitudinal and cross‐sectional studies (Britanova et al., 2014, 2016; Yoshida et al., 2017). As total T‐cell diversity declines proportionally to naive T‐cell numbers (Britanova et al., 2014), the decrease in diversity of the total CD8^+^ T‐cell pool is associated with decreased diversity in the naive CD8^+^ T‐cell pool (Egorov et al., 2018; Qi et al., 2014) (Figure 1). Indeed, it was shown that in aged mice the antigen‐specific T‐cell repertoire induced after antigen exposure was of lower diversity compared to that in young mice, which in turn was associated with lower numbers of naive precursor cells (La Gruta & Thomas, 2013; Quinn et al., 2016; Rudd et al., 2011; Smithey et al., 2012; Yager et al., 2008). In humans, diminished responses and narrower antigen‐experienced T‐cell repertoires have also been observed at older age, for example, in response to influenza A virus (IAV) infection (Deng et al., 2004; Murasko et al., 2002; Nguyen et al., 2017; Webster, 2000) or vaccination (Goronzy et al., 2001). If decreased diversity of the naive T‐cell pool indeed forms the bottleneck for the induction of protective memory T‐cell responses in older adults, instead of vaccinating elderly people, it may be better to induce protective immune responses by vaccination earlier in adulthood. It would then be essential that such immune responses are maintained well into old age. Little is known about how long antigen‐specific T cells are well maintained in the memory pool of older adults. Some studies have suggested that the diversity as well as the absolute number of cells in the CD8^+^ memory T‐cell compartment remain relatively stable with age (Qi et al., 2014; Wertheimer et al., 2014). This is in fact surprising for two reasons: (1) the memory pool is constantly fed with new T cells after every new antigen encounter and (2) memory T cells that reencounter their antigen, like chronic viruses, expand and thereby add cells to the memory T‐cell pool. It has been suggested that with every Tcell entering the memory pool, antigen‐experienced T cells already present in the memory pool may be eliminated (Figure 1), leading to memory attrition (Sad & Krishnan, 2003; Welsh & Selin, 2009). Especially cytomegalovirus (CMV) is known to frequently reactivate and challenge the immune system, thereby maintaining high frequencies of antigen‐specific T cells (Sinclair & Sissons, 2006; Vescovini et al., 2007; Weltevrede et al., 2016). The presence of these CMV‐specific clonal expansions has been proposed to lead to changes in the antigen‐specific T‐cell repertoire against other viruses (Jergovic et al., 2019) and could therefore affect the maintenance of other antigen‐specific T‐cell clones in the memory pool.

Box 1 **Challenges of studying T‐cell receptor diversity.**

T‐cell repertoire diversity is an important determinant of the efficacy of the CD8^+^ T‐cell response. However, determining the diversity of the T‐cell repertoire is challenging at various levels, making the comparison between T‐cell repertoire studies even more challenging. First, clinical samples used to determine T‐cell repertoire diversity represent only a tiny fraction of the total T‐cell pool present in the body. In theory, V(D)J recombination can result in more than 10^21^ unique T‐cell receptors. However, the number of different clonotypes remains unknown, but is estimated to lie between 10^7^ (Robins et al., 2009, 2010) and 10^10^ (Lythe et al., 2016). A clinical sample contains only a few milliliters of blood, while blood only contains 2 percent of all the T cells present in the body. This especially affects the identification of rare clonotypes (Robins et al., 2009).

Second, the differences in protocol used to estimate repertoire diversity can lead to discrepancies in conclusions. Especially when focusing on the antigen‐specific T‐cell repertoire, discrepancies are created by using different ways to detect enough antigen‐specific T cells, as back in the days the T‐cell population was often enriched through *ex vivo* stimulation. It was previously shown that repertoire analyses after *ex vivo* stimulation can lead to very different diversity measurements compared to direct ex vivo analyses (Koning et al., 2014). Thanks to the use of tetramers and single cell analyses, it becomes easier to determine the direct ex vivo repertoire of small cell populations. Also, the use of different techniques can result in discrepancies in conclusions, due to the level of depth that is accomplished. In the past, TCR‐Vβ antibodies were used to detect the presence of specific variable‐gene subgroups with in the β chain of the T‐cell receptor using flow cytometry. Nowadays, the usage of high‐throughput sequencing (HTS) is the norm, making it possible to analyze millions of different TCR sequences at once, mostly focusing on the β‐chain of the TCR. HTS provides much greater and more accurate quantification of the repertoire compared to the older techniques. However, also for HTS there are several available options and differences are seen throughout the whole protocol, for example, the starting material, PCR technique, and library preparation (Rosati et al., 2017). Furthermore, the data retrieved from HTS is very sensitive to PCR and sequencing errors. A step further in determining the diversity of the repertoire is the use of single cell analyses, which gives us more information on how the TCRα and TCRβ chain interact. Combining this with RNAseq analysis provides even more information, not only about the TCR usage, but also showing the functional state of T cells bearing a specific receptor. However, only a limited number of cells can be analyzed, making the diversity estimations difficult.

Last, diversity is still a poorly defined concept. One way to look at the diversity of a population is by looking at its richness and evenness. Richness is the actual number of unique TCR sequences in a population, whereas evenness reflects the relative frequencies of the different TCR sequences observed. The first measure is particularly sensitive to the number of rare clonotypes, which are easily overlooked when using small samples. Conversely, evenness is strongly affected by clonal expansions (Box Figure). For a good estimation of the diversity, both components are needed and are therefore integrated in several diversity indices, like Simpson diversity index or Shannon diversity index. Both indices are often used in the field; however, both have their own shortcomings and do not always properly reflect the repertoire distribution. For example, Simpson diversity index puts more weight on dominant species. This means that clonal expansions influence the outcome of this index largely and that a decrease in diversity may be seen, due to clonal expansions, while the richness of the population did not change. Shannon diversity index on the other hand, puts more weight on richness of the sample. Note that a high richness does not always implies a high diversity, as combined to a low evenness will lead to a low diversity (see Box Figure). Therefore, caution is needed when using diversity measurements, as diversity may be too complex to be noted by only one number (Johnson et al., 2014; Kaplinsky & Arnaout, 2016; Laydon et al., 2015; Yeom & Kim, 2011).

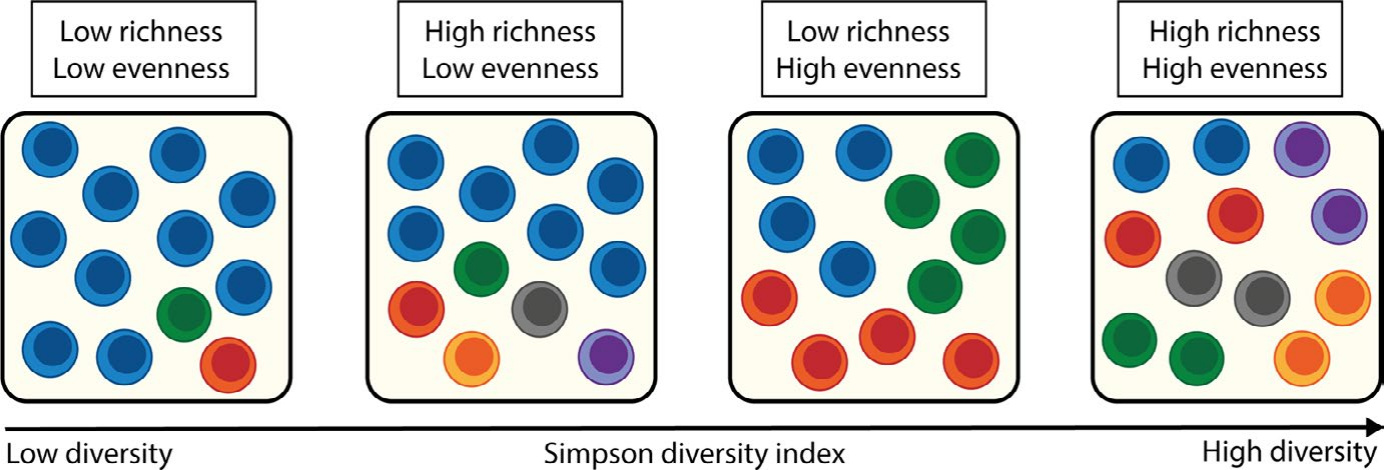

**Box Figure** **.** How richness and evenness make up the diversity. This figure shows how richness and evenness affect the diversity index when estimating the diversity of the repertoire. The four boxes show an increasing diversity, with varying diversity scores, based on Simpson diversity index. Note that the diversity indices of population 2 and 3 are very close, when diversity would be calculated with Shannon diversity index their order would be reversed.

This review provides an overview of studies addressing the maintenance of antigen‐specific CD8^+^ T‐cell repertoires in the memory pool. We address the influence of age on the dynamics of the antigen‐experienced T‐cell repertoire after infection. In addition, we cover how infection history and TCR affinity shape the antigen‐specific T‐cell repertoire. Finally, we discuss the implications of these findings for the development of vaccination strategies to protect older adults against infectious diseases.

## 2 CHANGES IN THE ANTIGEN‐SPECIFIC T‐CELL REPERTOIRE WITH AGE

To assess how the antigen‐specific T‐cell repertoire in the memory pool changes with age, studies that analyze single epitope‐specific repertoires are needed. Most antigen‐specific studies in humans have focused on CD8^+^ T cells directed against epitopes of influenza virus and the chronic viruses CMV and Epstein–Barr virus (EBV). Antigen‐specific T cells against these viruses are present in relatively high frequencies in the blood, making them easily detectable by tetramer staining or peptide stimulation. There is also quite a lot of literature on T‐cell repertoire diversity in HIV infection, however, as the interpretation of these data is complicated by the high mutation rate of the virus, heterogenicity in rates of disease progression and the use of antiviral therapy, we here focus on the antigen‐specific response to the herpesviruses CMV and EBV and the memory phase of influenza virus‐specific T cells during homeostatis.

### 2.1 The CMV‐specific T‐cell repertoire

CMV is a herpesvirus, known for its latent presence in the body. The virus is thought to frequently reactivate, leading to restimulation of the anti‐CMV immune response. Hallmarks of CMV infection are the presence of clonal expansions and large numbers of highly differentiated cells in the CD8^+^ T‐cell memory pool (van den Berg et al., 2019). These clonal expansions are often directed to the pp65 protein and can be focused on only one or a small number of epitopes, like the HLA‐A2 restricted NLVPMVATV (A2‐NLV) epitope. The antigen‐specific T‐cell repertoire against the A2‐NLV epitope has already been described more than two decades ago using TCR‐Vβ antibodies, recognizing the different variable segments of the β‐chain of the T‐cell receptor (Box 1). This revealed that the T‐cell response against A2‐NLV shows preferential usage of Vβ8 and Vβ17 (Weekes et al., 1998; Wills et al., 1996). Skewed Vβ usage in the CMV‐specific T‐cell repertoire has also been observed in other studies (Attaf et al., 2018; Trautmann et al., 2005; Wynn et al., 2008) and is not restricted to the pp65 antigen (Cardenas Sierra et al., 2014; Khan et al., 2002; Lim et al., 2000; Miles et al., 2005; Weekes et al., 1998). Sequencing of cultured A2‐NLV‐specific T‐cell clones revealed the presence of the same clones at different time points over a time span of 18 months (Weekes et al., 1998). When these results were compared to a group of older donors (Khan et al., 2002), notable similarities were observed, including similar skewing toward Vβ8 segment usage and even the occurrence of the same clonotypes, also known as public clonotypes. It was suggested by both studies that CMV‐specific T‐cell clones can be maintained during life.

A later study suggested that the repertoire is less stable than originally thought. In fact, within the A2‐NLV Vβ8^+^ T‐cell repertoire, the clonotype usage turned out to be much less stable than previously anticipated (Schwanninger et al., 2008). One T‐cell clone was detected in 72% of the sequences in young donors (28–37 years), while its dominance decreased to below 40% in middle‐aged and older adults. Interestingly, the TCR of another dominant clone that was only observed in older adults had a higher affinity for the A2‐NLV epitope than the clone dominating in young donors (Schwanninger et al., 2008). This suggests that at older age, higher avidity T‐cell clones become more dominant. Following the CMV‐specific T‐cell repertoire after primary infection showed that in the memory phase high avidity clones became dominant, which were subdominant early after primary infection (Day et al., 2007). Together this suggests that there is a shift in clonal dominance based on TCR affinity, even though the biased Vβ segment usage in the CMV‐specific T‐cell repertoire seems to be maintained over time.

Longitudinal studies have suggested that a large part of the A2‐NLV‐specific T‐cell repertoire is conserved for at least 2 (Hadrup et al., 2006) to 4 years (Iancu et al., 2009). 60%–100% of the clones found in middle‐aged and older adults persisted over a period of 2 years (Hadrup et al., 2006). A more in‐depth analysis of the CMV‐specific T‐cell repertoire after 4 years showed that a large proportion of the T‐cell repertoire was stable over time, with persistence of the most dominant T‐cell clones (Iancu et al., 2009). This stable repertoire has been suggested to be formed very early after primary infection, as the responding T‐cell repertoires 6–16 weeks after infection were comparable to those in the same donors 3–5 years later (Klarenbeek et al., 2012). Although these longitudinal studies have suggested that the antigen‐specific T‐cell repertoire is relatively stable over time, the time frames of these studies are probably too short to show a shift in the T‐cell repertoire as suggested by cross‐sectional studies. In summary, the CMV‐specific T‐cell repertoire seems to consist of persistent T‐cell clones, at least for relatively short periods of time, while with age, shifts in dominance may very well occur, probably selected based on their TCR affinity.

### 2.2 The EBV‐specific T‐cell repertoire

Although both CMV and EBV are chronic herpesviruses, known for their latent presence in the body, they have their own dynamics when it comes to infection and reactivation. EBV has a more restricted cell tropism than CMV (Hatton et al., 2014; Shenk & Stinski, 2008) and is thought to reactivate less often (Scheinberg et al., 2007; Thomasini et al., 2017). Although EBV‐specific T‐cell frequencies are also relatively high, T‐cell expansions specific for EBV are less pronounced than for CMV (Khan et al., 2004; Sukdolak et al., 2013). Whether these differences also lead to different T‐cell repertoire diversities for these two viruses remains debated. The T‐cell repertoire against the frequently studied HLA‐A2 restricted EBV epitope GLCTLVAML (A2‐GLC) tends to be broader than the repertoire recognizing the A2‐NLV epitope of CMV (Iancu et al., 2009). However, some similarities are observed between the repertoires, as both are oligoclonal, highly skewed and containing public T‐cell clones (Price et al., 2005). Furthermore, like the CMV‐specific T‐cell repertoire, the EBV (A2‐GLC)‐specific T‐cell repertoire is characterized by dominant usage of particular Vβ segments, in this case Vβ20‐1 and Vβ29‐1 (previously known as Vβ2 and Vβ4) (Iancu et al., 2009, 2013; Lim et al., 2000).

Several studies have suggested that the EBV‐specific T‐cell repertoire is very stable. For example, the A2‐GLC‐specific T‐cell repertoire recovered in melanoma patients after transient lymphocyte depletion showed a similar bias in Vβ‐segment usage as the A2‐GLC‐specific T‐cell repertoire in healthy donors, including the same dominant T‐cell clonotypes (Iancu et al., 2013). This suggests that once the EBV‐specific T‐cell repertoire is established, its clonal composition remains rather stable. In line with this, in a patient undergoing primary EBV infection after renal transplantation, the repertoire against two HLA‐B35 restricted epitopes was highly stable from 18 weeks after infection up to 3 years later (Klarenbeek et al., 2012). However, it was suggested that the clones that were detected during the chronic phase were not the most dominant clones during the peak of the primary infection. Sequencing A2‐GLC‐specific T‐cell clones early after primary infection (7 till 15 days) and 2 years later actually showed, based on Vβ usage, very different clonotypes dominating the primary and the memory response (Annels et al., 2000). These data thereby suggest that the T‐cell response against EBV changes during the first phase of the infection, to become stable in the chronic phase up to at least 3 years after infection.

In contrast to the stable Vβ usage observed in longitudinal studies, a cross‐sectional study reported differences in Vβ‐segment usage between the EBV‐specific T‐cell repertoire of young and older adults. Although both age groups had a polyclonal and diverse TCR‐Vβ repertoire specific for EBV, in the older adults the frequency of TCRs from the Vβ9 family was higher, while in the younger adults, T‐cell clones from the Vβ13‐1 family dominated (Cardenas Sierra et al., 2014). In summary, the EBV‐specific T‐cell repertoire shows a relatively stable usage of Vβ segments after the peak of the primary response.

### 2.3 The influenza virus‐specific T‐cell repertoire

Although influenza A virus (IAV) infection is an acute infection, reinfection may occur several times during life (Francis et al., 2019; Lessler et al., 2012). The effect of age on the IAV‐specific T‐cell repertoire has been studied extensively, as there is an age‐associated increase of complications due to IAV infection (McElhaney et al., 2016). Although IAV is known for the high mutation rate of its surface proteins, the internal proteins are relatively stable, leading to restimulation of IAV‐specific CD8^+^ T cells. The best studied IAV‐specific epitope is the HLA‐A2 restricted GILGFVFTL epitope (A2‐GILG), present in the M1 protein that is highly conserved between different IAV strains (Gianfrani et al., 2000).

The A2‐GILG‐specific T‐cell repertoire shows a more skewed distribution based on Vβ‐usage compared to the CMV A2‐NLV‐specific and EBV A2‐GLC‐specific T‐cell repertoires. This may in part be explained by the reduced accessibility of the GILG epitope when presented by the HLA‐A2 molecule (Chen et al., 2017). Up to 90% of the A2‐GILG‐specific T cells express the Vβ19 segment and a conserved arginine‐serine motif, also referred to as the “RS‐motif”, in the CDR3 region of the responding T cells is often observed (Lehner et al., 1995; Moss et al., 1991; Stewart‐Jones et al., 2003). Because such a large fraction of TCRs express the Vβ19 segment, several T‐cell repertoire studies have focused on the dynamics of the Vβ19^+^ T‐cell clones within the A2‐GILG‐specific T‐cell repertoire. One study reported a decrease in diversity when comparing the A2‐GILG Vβ19^+^ T‐cell repertoires of middle‐aged and older adults using different diversity measures (Gil et al., 2015). A comparable study, on the other hand, did not find a significant loss of GILG‐specific T‐cell diversity with age. Although TCR richness was lower in older adults, the overall diversity of the repertoire was not. It was suggested that the overall diversity (which takes into account both richness and evenness) of the responding repertoire did not decrease with age, because loss in richness was counterbalanced by increased evenness of the repertoire (Naumov et al., 2011) (see Box 1).

A longitudinal study among 3 healthy volunteers over a time span of 7–10 years showed that many characteristics of the A2‐GILG Vβ19^+^ T‐cell repertoire were very stable over 7–10 years (Naumova et al., 2009): identical clonotypes were found, the repertoire always showed the same level of skewing and diversity indices of the A2‐GILG‐specific T‐cell repertoire did not change significantly. Nevertheless, some shifts within the Vβ19^+^ T‐cell clones were observed, as there was a loss of clonotypes with the RS‐motif with age (Naumova et al., 2009).

Also for the TCRα chain, dominance of certain V‐segments (i.e., Vα12 and Vα27, often expressed in combination with the Vβ19 segment) has been observed in the IAV‐specific T‐cell repertoire (Gil et al., 2015; Sant et al., 2018). The A2‐GILG‐specific T‐cell repertoires of older adults showed more dominant usage of the Vα27 segment and less dominant usage of the Vα12 segment than those of middle‐aged adults. However, within the Vα27^+^ IAV‐specific T‐cell repertoire, TCR richness decreased with age. Thus, also the TCRα usage of the IAV‐specific T‐cell repertoire changes considerably over time.

Remarkably, studies focusing on the complete A2‐GILG‐specific T‐cell repertoire and those focusing on the Vβ19^+^ repertoire have reached different conclusions. Studies focusing only on the Vβ19 expressing GILG‐specific T‐cell repertoire seem to miss a large part of the responding repertoire, especially in older individuals. Although almost all T‐cell receptors against A2‐GILG in young adults expressed the Vβ19 segment, in older adults this was only the case for 55% of the responding TCRs. A similar decrease was observed for the expression of the conserved “RS‐motif” (Nguyen et al., 2017). Direct *ex vivo* paired analyses of the TCRα and β chains of the A2‐GILG‐specific T‐cell repertoire showed that older adults tended to have a reduced TCR diversity within the Vβ19^+^ clonotypes, while their more private/non‐Vβ19^+^ T‐cell receptors consisted of a broader usage of Vβ and Vα segments compared to young adults (Nguyen et al., 2017; Sant et al., 2018). Despite these differences in TCR usage, the *in vitro* proliferative capacity of the A2‐GILG‐specific T‐cell repertoires of young and old adults was comparable after peptide stimulation (Nguyen et al., 2017). Thus, Vβ19^+^ T‐cell clones recognizing IAV become less dominant in older adults, leading to a more diverse usage of other Vβ segments within the IAV‐specific T‐cell repertoire.

Because the A2‐GILG‐specific T‐cell repertoire has been analyzed in so much detail, the data also allow to study changes of other TCR characteristics, such as CDR3 length and amino acid usage of the TCR chains. In general, a characteristic of the A2‐GILG‐specific T‐cell repertoire is the 11 amino acid length of the CDR3 loops. Interestingly, it was shown that this characteristic becomes less prominent with age, giving rise to a broader range of longer CDR3 loops (Gil et al., 2015; Nguyen et al., 2017; Sant et al., 2018). Furthermore, in older adults, the frequencies of glycines and alanines in the CDR3 region of the A2‐GILG‐specific T‐cell repertoire appeared to be higher than in middle‐aged persons. The usage of more alanines and glycines and longer CDR3 loops is thought to lead to a more flexible CDR3 region resulting in a more cross‐reactive T‐cell receptor (Naumov et al., 2008; Welsh et al., 2010). This would suggest that the A2‐GILG‐specific T‐cell repertoire would be more cross‐reactive in older adults, which could lead to better recognition of escape variants of the epitope (Petrova et al., 2012), although these are not expected to occur very often, considering the stable nature of the epitope A2‐GILG epitope.

In conclusion, Vβ19 skewing of the A2‐GILG‐specific T‐cell repertoire is mainly present in young adults and seems to be less prominent in older adults, leading to a more diverse IAV‐specific T‐cell repertoire with age. However, especially in the case of IAV, it is unknown whether the loss of dominant T‐cell clones is due to aging of the immune system, or whether restimulation due to reinfection plays an important role in the formation of the T‐cell repertoire. It is therefore also important to obtain insights into the effect of infection history on the composition of the antigen‐specific T‐cell repertoire.

## 3 EFFECTS OF INFECTION HISTORY ON THE ANTIGEN‐SPECIFIC T‐CELL REPERTOIRE

It is generally thought that homologous infection can lead to selective expansion of antigen‐specific T cells. How the antigen‐specific repertoire is shaped after repetitive stimulation is not completely understood, but the TCR affinity for the peptide‐MHC complex is thought to play an important role. Next to homologous infections, also heterologous infections may influence the antigen‐specific T‐cell repertoire. The effect of such heterologous infections on the antigen‐specific T‐cell repertoire likely depends on the degree of antigenic similarity between infections, although it has also been described for infections with very minimal (Oberle et al. 2016) or even no overlap (Selin et al., 1999; Smithey et al., 2018). When investigating the maintenance of the antigen‐specific T‐cell repertoire with age, it is therefore important to understand how its diversity is influenced by both homologous and heterologous infections.

### 3.1 Homologous virus infections

**Figure 2:**
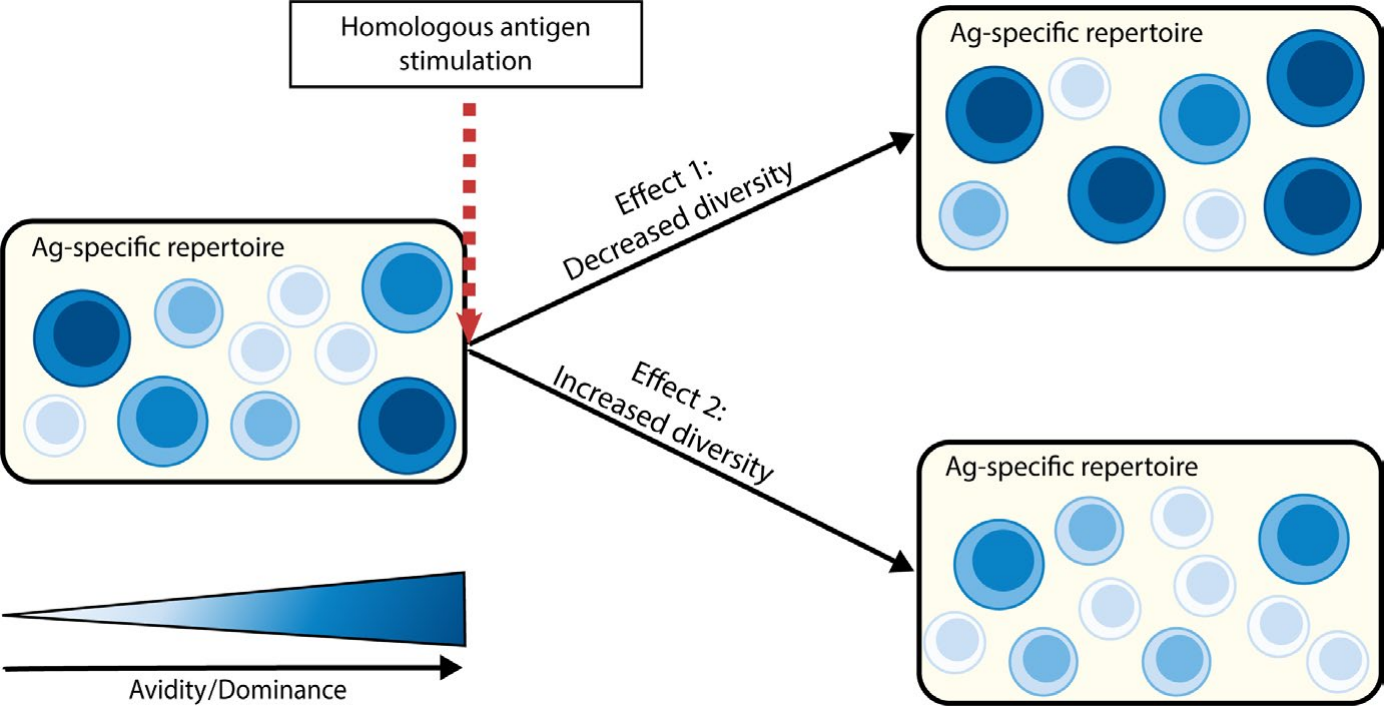
Possible effects of homologous antigen stimulation on the antigen‐specific T‐cell repertoire. This figure shows two (quite opposing) effects that may impact the composition of the antigen‐specific T‐cell repertoire after homologous antigen stimulation. Size of circles indicates clonal size, darkness indicates avidity/dominance of the clones. Effect 1 refers to the selection of the most dominant, high avidity clones after stimulation, leading to a skewed antigen‐specific T‐cell repertoire, while effect 2 refers to the loss of the most dominant, high avidity clones, leading to a more diverse antigen‐specific T‐cell repertoire

Restimulation of the immune system by the same virus can occur both for acute and chronic infections, by reinfection or reactivation, respectively. It is widely accepted that during primary infection, T‐cell clones expressing high‐affinity TCRs tend to be selected to respond. The effect of repetitive antigen stimulation on the T‐cell repertoire is however still debated, and in fact two opposing effects have been proposed (Figure 2):

1. A decrease in diversity and increased skewing of the antigen‐specific T‐cell repertoire, as mainly dominant, high avidity clones in the repertoire may expand upon antigen reencounter (Busch & Pamer, 1999; Savage et al., 1999).
2. An increase in diversity of the antigen‐specific T‐cell repertoire, due to overstimulation and subsequent loss of the most dominant high avidity clones, leading to an increased representation of clones that used to be subdominant. (Davenport et al., 2002; Naumov et al., 2011; Vigano et al., 2012).

The first effect, selection of high avidity clones upon antigen reencounter, has been observed in an acute *Listeria monocytogenes* infection model in mice, where a more focused antigen‐specific T‐cell population emerged after a second challenge (Busch et al., 1998). The new antigen‐specific T‐cell pool appeared to have a higher avidity for *the L*.*monocytogenes*‐specific peptide and a more intense tetramer staining compared to the T‐cell population after primary infection (Busch & Pamer, 1999). A highly focused T‐cell repertoire consisting of high avidity T‐cell clones has also been observed for the CMV‐specific T‐cell repertoire in humans. Again, the highly focused A2‐NLV‐specific T‐cell repertoire was due to the presence of dominant clones with a relatively high TCR affinity for the peptide (Trautmann et al., 2005). In line with this, another study found that the most differentiated and expanded T‐cell clones specific for CMV and EBV were the clones with the highest avidity (Price et al., 2005).

In contrast, some other findings support the view that clonal selection of low‐avidity clones through loss of high avidity clones (effect 2) occurs in both humans and mice (Schober et al., 2020) in the context of chronic CMV infection. In humans, a negative correlation was observed between the frequencies of TCRs recognizing the HLA‐B8 QIKVRVDMV (B8‐QIK) CMV epitope and their affinity. In mice infected with an MCMV strain expressing the H2K^b^‐SIINFEKL epitope under an MCMV inflationary promotor (IE2), high avidity T‐cell clones had a more senescent phenotype. The authors proposed that selection of low‐affinity TCRs is due to the chronic nature of the virus, which induces a senescent phenotype of the high avidity clones (Schober et al., 2020). While they propose that in acute infections, clonal selection favors T‐cell clones with high avidity. It remains unclear why even for a single pathogen such opposing results have been reported, and to what extent the use of different epitopes, and the frequency of viral reactivation could contribute to these differences.

The results of Schober *et al*. are in line with the basis of a modeling study, which proposed that repetitive stimulation due to virus reactivation could lead to loss of high avidity clones (Davenport et al., 2002). *Ex vivo* analyses showed that during primary EBV infection, high‐affinity EBV‐specific TCRs were selected, while 1 year later a decrease in affinity was observed. Clones that were less likely to expand during primary infection contributed more to the repertoire 1 year later (Davenport et al., 2002). To explain these observed effects, factors such as cellular senescence needed to be introduced in the model, suggesting that loss of previously dominant high avidity clones through senescence may play an important role in shaping the T‐cell repertoire. A comparable result was described for the IAV Vβ19^+^ A2‐GILG‐specific T‐cell repertoire, as *ex vivo* lower diversity was observed in older compared to middle‐aged individuals. Model analysis of these data showed that the parameters explaining the *ex vivo* results included a combination of senescence and selective expansion of clones (Naumov et al., 2011), leading to a repertoire that progressively consists of clonotypes that used to be less dominant.

Analysis of the effects of viral reencounter in a more controlled way in mice has suggested that the antigen‐specific T‐cell repertoire remains relatively stable. A frequently used model in mice is repetitive infection with the (acute) lymphocyte choriomeningitis virus (LCMV) Armstrong strain. The T‐cell repertoire against LCMV has a preferential usage of certain Vβ segments, including Vβ8.1 and Vβ8.2. The recall response consisted of large expansions of LCMV‐specific T cells and a very similar skewing in Vβ usage was observed after primary and secondary infection. Based on these findings, it was suggested that the LCMV‐specific T‐cell repertoire remained relatively stable (Blattman et al., 2000; Lin & Welsh, 1998; Sourdive et al., 1998). Epitope dominance seemed to play a considerable role in shaping the LCMV‐specific response, as after secondary infection a profound shift in epitope immunodominance was observed (Blattman et al., 2000). These data suggest that in the case of LCMV infection after at least 1 recall challenge, no specific (high avidity) clone selection can yet be detected.

Also repetitive infection of mice with IAV generally shows that the levels of diversity of the responding T‐cell repertoires after primary and secondary infection are comparable (Flynn et al., 1998; Kedzierska et al., 2006; Turner et al., 2003). These studies included a more detailed analysis of the antigen‐specific repertoire compared to studies on LCMV infection and therefore differences in clonal composition after primary and secondary IAV infection could be revealed. Some clones were found after both infections and others were only found after primary or secondary infection (Turner et al., 2003). Transferring antigen‐experienced influenza virus‐specific (Db‐NP366) Vβ13^+^ T cells into mice, followed by IAV infection led to an increase of one particular clone with a relatively low start frequency. As a consequence, the frequency of one or two clonotypes that were dominant in the first mouse reduced (Cukalac et al., 2014). This effect is somewhat comparable to what is observed for the A2‐GILG‐specific repertoire in older adults, where a loss of clones consisting of the public “RS‐motif” is observed within the dominantly used Vβ segment (Gil et al., 2015). These data suggest that changes that tend to be described to aging in humans may also be due to repetitive stimulation.

In conclusion, which affinity‐based mechanism mostly occurs *in vivo* after homologous antigen stimulation remains unclear. The dominant mechanisms may be different per virus and epitope, as experimental evidence has been found for both effects even for the same virus. Apart from affinity‐based mechanisms, more recently it has been suggested that stochastic expansion of T cells may contribute to the maintenance of T‐cell diversity in the memory pool. It was shown in mice that different MCMV‐specific clonotypes underwent proliferation irrespective of their avidity, leading to a stable repertoire up to a year postinfection. It was suggested that T‐cell proliferation occurs in a stochastic manner, leading to the maintenance of both dominant and subdominant clonotypes, thereby contributing to a diverse antigen‐specific repertoire (Smith et al., 2020).

### 3.2 Heterologous virus infections

The immune response induced by a previously encountered pathogen can even alter the immune response to new unrelated virus infections, a phenomenon also known as heterologous immunity (Selin et al., 1999). In the light of heterologous immunity, infection with CMV has been of particular interest, because of the notoriously large clonal expansions it induces. Percentages of CMV‐specific T cells up to 40% of the total memory T‐cell compartment have been reported (Khan et al., 2002; Remmerswaal et al., 2015). One of the first studies investigating the influence of CMV on the total CD8^+^ T‐cell repertoire, showed that the number of clonal expansions in CMV^+^ individuals was more than 30% higher than in CMV^−^ donors (Khan et al., 2002). Even though the specificity of these clonal expansions was not determined, the effect of CMV serostatus on the diversity of the memory T‐cell pool was evident (Khan et al., 2002). Later studies showed that the expanded clones in the memory T‐cell pool of CMV^+^ individuals were CMV‐specific (Emerson et al., 2017). Therefore, it has been suggested that CMV infection may also influence T‐cell repertoire diversity, especially within the memory T‐cell population (Jergovic et al., 2019).

In the last decades, the theory that CMV infection would have a negative influence on unrelated T cells, dominated the field. The large CMV‐specific clonal expansions were thought to outcompete other T cells by competing for growth and survival factors (Derhovanessian et al., 2009; Pawelec et al., 2005; Tu & Rao, 2016). This hypothesis was supported by the finding that a decline in T‐cell repertoire richness with age is only observed in the memory T‐cell population of CMV^+^ individuals (Qi et al., 2014). One study so far attempted to compare the diversity of the non‐CMV‐specific memory T‐cell pools of 550 donors, either CMV^+^ or CMV^−^, by leaving out the most numerous 0.1% of the clones in the memory compartments from the analysis. These clones were removed as they were clearly larger in CMV^+^ compared to CMV^−^ individuals, and for a few donors it was shown that these clones reacted to CMV‐specific antigens. The diversity scores of the remaining “non‐CMV‐specific” memory T‐cell pools of CMV^+^ and CMV^−^ individuals turned out to be comparable (Lindau et al., 2019). This study suggests that the T‐cell pool accommodates large CMV‐specific T‐cell clones by simply expanding and that CMV‐specific clonal expansions do not compromise the CD8^+^ T‐cell repertoire against other antigens.

To asses the impact of previous CMV infection on the diversity of the T‐cell response to a new unrelated pathogen, the OVA‐specific Vβ12^+^ T‐cell repertoire in response to Listeria infection expressing ovalbumin was compared between MCMV^+^ and MCMV^−^ mice. An unexpected positive effect was observed: the diversity of the OVA‐specific T‐cell repertoire in aged MCMV^+^ mice turned out to be higher than in aged MCMV^−^ mice. How this more diverse repertoire was established in the MCMV^+^ mice remains unclear. Interestingly, sequencing of the naive T‐cell repertoires of the mice showed comparable diversity, suggesting that similar numbers of precursor T cells were available (Smithey et al., 2018). The more diverse OVA‐specific T‐cell repertoire in MCMV^+^ mice also led to a better IFNγ response to OVA peptide stimulation than in MCMV^−^ mice. These findings are surprising in light of earlier studies, which showed a significantly weaker response against heterologous infections, including LCMV and IAV, following MCMV infection (Cicin‐Sain et al., 2012; Redeker et al., 2017; Smithey et al., 2012). If anything, these data emphasize the complicated influence of CMV infection on the T‐cell pool.

Heterologous immunity can also occur for virus infections that get cleared and nevertheless influence the antigen‐specific T‐cell repertoire to other pathogens. In LCMV‐infected mice, the LCMV‐specific T‐cell repertoire is typically strongly skewed toward expression of Vβ8.1, which is also observed after secondary LCMV infection (Blattman et al., 2000). However, infection of mice with LCMV, followed by infection with pichinde virus (PV) and vaccinia virus (VV), and then followed by secondary LCMV infection, led to reduced frequencies of LCMV‐specific T cells and to an altered usage of Vβ segments (Selin et al., 1999). Effects of PV infection on LCMV infection have been observed more often, and it has been suggested that PV infection leads to a narrowed LCMV‐specific repertoire (Selin 1994; Cornberg et al., 2010; Welsh et al., 2010). This heterologous immunity is probably due to the relatively high level of sequence similarity between the different virus‐specific epitopes, causing some TCRs to recognize both epitopes, also known as cross‐reactivity.

In humans, cross‐reactivity has been observed for the EBV‐specific A2‐GLC and IAV‐specific A2‐GILG epitopes, epitopes which only have 3 amino acids in common, however most of the involved TCRs showed to have a low affinity for both peptides (Clute et al., 2005; Cornberg et al., 2010; Watkin et al., 2017). It has been suggested that cross‐reactivity between antigens may narrow the responding T‐cell repertoire, as the subset of T cells already present in the memory pool specific to one epitope can grow out in response to a new epitope, thereby limiting the recruitment of new naive T cells into the memory T‐cell pool (Welsh et al., 2010). On the other hand, as has been proposed for homologous infections, one could argue that heterologous infections may lead to a decrease or an increase in the diversity of the antigen‐specific repertoire. It was shown by computer simulation that the affinity of the TCR is probably an important factor determining whether cross‐reactivity leads to broadening or narrowing of the antigen‐specific T‐cell repertoire. The more dissimilar the epitopes, as seen for A2‐GLC and A2‐GILG for example, the more likely broadening of the repertoire would occur, whereas if the epitopes are more similar, as seen for LCMV and PV, narrowing of the repertoire would be a more likely outcome (Clute et al., 2010).

In conclusion, for both homologous and heterologous infections an influence on the antigen‐experienced T‐cell repertoire can be expected. It remains unclear, however, whether a positive or negative effect will occur. Furthermore, whether the effects observed in mice are comparable to those occurring in humans remains unknown. Differences can be due to the infection order. For example, mice are typically infected with MCMV long before they receive a heterologous infection, while humans gradually build up memory against other infections, making it much more challenging to study the effect of CMV in the human setting.

## 4 IMPLICATIONS FOR VACCINE STRATEGIES IN OLDER ADULTS

It is generally assumed that the diversity of the naive T‐cell repertoire in elderly people is one of the limiting factors for the induction of T‐cell responses to new antigens. Vaccinating at an earlier age during adulthood may lead to better responses at older age, but to protect the older adults it is essential that such T‐cell clones are maintained over time. Therefore, in this review we investigated (1) the effect of age on the antigen‐specific repertoire, (2) the impact of repetitive stimulation on the antigen‐specific T‐cell repertoire, and (3) the influence of heterologous immunity, especially CMV infection, on the T‐cell repertoire against other antigens.

The antigen‐specific repertoire dynamics in the memory pool described for CMV, EBV, and IAV suggest that the repertoires against EBV and CMV are stable for at least a couple of years, while for IAV a decrease of the most dominant TCRs is observed with age. In addition to age, infection history seems to be a key player shaping the repertoire dynamics against these three viruses over time. Studying the maintenance of the T‐cell repertoire against an infection we only encounter once could provide better insight into the longevity of the immune response. A frequently used model for an acute challenge in humans is yellow fever (YF) vaccination. This live‐attenuated vaccination is thought to be one of the most effective vaccines currently available for the induction of T cells (Co et al., 2009; DeWitt et al., 2015), as even decades later YF‐specific T cells can still be observed in the blood (Wieten et al., 2016). However, data on the dynamics of the T‐cell repertoire against YF over a longer time period remain limited. In one donor, most of the clones identified after the booster vaccination were comparable to the clones observed after the primary infection 18 months earlier. In another donor who received the first vaccination 30 years earlier, it was shown that most YF‐specific clonotypes were of low frequency or undetectable when sequencing the bulk memory T‐cell pool. Shortly after booster vaccination, however, expansion of YF‐specific T‐cell clones was observed via sequence analysis, suggesting that YF‐specific T cells remained present for 30 years (Minervina et al., 2020).

Based on the available data, the effect of repetitive stimulation on the repertoire remains inconclusive, as both expansion and loss of dominant, high avidity clones has been observed. The effect of homologous restimulation is interesting in the light of booster vaccinations and vaccinations that are regularly given (e.g., seasonal flu vaccination), as these induce comparable repetitive T‐cell antigen stimulation. This may be a vaccine strategy to overcome diminished responses at older age. However, it remains unknown when boosting results in the maintenance of a diverse repertoire or in the expansion of high‐affinity clones, and in fact, it is not even known which of these outcomes would be favorable. Although the hypothesis of Schober *et al*. (Schober et al., 2020) that discrepancies between studies are due to the chronic or acute nature of the viruses is very attractive, such discrepancies are even observed between studies focusing on acute or chronic infections only.

Heterologous infections could also lead to a decrease or increase in the diversity of unrelated T‐cell repertoires. Especially the effect of CMV infection on the memory response to unrelated antigens should be studied in more detail in humans, as CMV occupies a large part of the memory T‐cell pool. Given that CMV‐specific T‐cell responses are maintained at such high frequencies with age, CMV is regarded as a promising candidate to use as viral vaccine vector. The relative stability of the CMV‐specific repertoire is an additional advantage for using CMV as a vector. Although the developments are still at an early stage, studies using CMV as viral vaccine vector against HIV, SIV, and tuberculosis show broad and robust T‐cell responses (Liu et al., 2019).

Other factors apart from aging and infection history may also play an important role in the dynamics and stability of antigen‐specific T‐cell repertoires, including the type of antigen, the processing and presentation of the antigen (Kotturi et al., 2008), the dose of the antigen (Naumov et al., 2011), and the recruitment of T cells to the site of infection. The relative impact of these factors on the resulting T‐cell repertoire still has to be determined. Nevertheless, alternative vaccination strategies linked to these factors have been proposed to improve the CD8^+^ T‐cell response in elderly, including the use of stronger adjuvants, higher antigen doses (McElhaney, 2011; Weinberger, 2018a, 2018b) or modified pMHC interactions (Rosendahl Huber et al., 2016). However, these strategies may in turn exert an effect on the responding T‐cell repertoire. It was indeed shown in mice that the use of different adjuvants can alter the composition of the T‐cell repertoire, due to changes in affinity thresholds for TCR selection (Malherbe et al., 2008).

Although previous studies provided insight in T‐cell repertoire dynamics, several questions remain: How to link diversity measurements to T‐cell efficacy? Why do certain T‐cell specificities survive while others disappear? And are certain TCRs needed to elicit a protective immune response? In line with these questions, there is a lot of interest for public T‐cell clones and the preferred usage of certain Vβ segments, assuming that these provide an evolutionary selective advantage (Miles et al., 2011). It has been proposed that certain Vβ segments provide a more favorable interaction between the TCR and the pMHC complex (Chen et al., 2017; Gras et al., 2008). It remains unclear, however, to what extent the occurrence of public TCRs can be explained by their functional role in the recognition of certain epitopes, and to what extent they have a higher generation probability during VDJ recombination (Elhanati et al., 2018; Venturi et al., 2006, 2008). Finally, there is a need for long‐term longitudinal studies, mostly to figure out basic questions like: How long is repertoire diversity to a new antigen maintained? How is it maintained after reexposure? And is it maintained in combination with other unrelated infections?

## 5 CONCLUSION

Knowledge on the dynamics of the antigen‐specific T‐cell repertoire could lead to important insights that can be used to optimize vaccine strategies to protect older adults. Although some challenging questions still need to be answered, important steps have been made in the past years. The first studies investigating how to identify an individual's infection history by dissecting the antigen‐specific T‐cell repertoire using antigen‐specific TCR motifs are very promising (Dash et al., 2017; Glanville et al., 2017). Such information could potentially lead to the design of a more personalized vaccination program, using alternative vaccination strategies including earlier vaccination to optimize T‐cell responses at older age.

## CONFLICT OF INTEREST

The authors declare to have no conflict of interest.
